# Genomic and Resistance Gene Homolog Diversity of the Dominant Tallgrass Prairie Species across the U.S. Great Plains Precipitation Gradient

**DOI:** 10.1371/journal.pone.0017641

**Published:** 2011-04-12

**Authors:** Matthew N. Rouse, Amgad A. Saleh, Amadou Seck, Kathleen H. Keeler, Steven E. Travers, Scot H. Hulbert, Karen A. Garrett

**Affiliations:** 1 Department of Plant Pathology, Kansas State University, Manhattan, Kansas, United States of America; 2 School of Biological Sciences, University of Nebraska, Lincoln, Nebraska, United States of America; University of Chicago, United States of America

## Abstract

**Background:**

Environmental variables such as moisture availability are often important in determining species prevalence and intraspecific diversity. The population genetic structure of dominant plant species in response to a cline of these variables has rarely been addressed. We evaluated the spatial genetic structure and diversity of *Andropogon gerardii* populations across the U.S. Great Plains precipitation gradient, ranging from approximately 48 cm/year to 105 cm/year.

**Methodology/Principal Findings:**

Genomic diversity was evaluated with AFLP markers and diversity of a disease resistance gene homolog was evaluated by PCR-amplification and digestion with restriction enzymes. We determined the degree of spatial genetic structure using Mantel tests. Genomic and resistance gene homolog diversity were evaluated across prairies using Shannon's index and by averaging haplotype dissimilarity. Trends in diversity across prairies were determined using linear regression of diversity on average precipitation for each prairie. We identified significant spatial genetic structure, with genomic similarity decreasing as a function of distance between samples. However, our data indicated that genome-wide diversity did not vary consistently across the precipitation gradient. In contrast, we found that disease resistance gene homolog diversity was positively correlated with precipitation.

**Significance:**

Prairie remnants differ in the genetic resources they maintain. Selection and evolution in this disease resistance homolog is environmentally dependent. Overall, we found that, though this environmental gradient may not predict genomic diversity, individual traits such as disease resistance genes may vary significantly.

## Introduction

Intraspecific variation has been proposed to vary across space according to peak or gradient models [1]. In the peak model, species traits such as diversity vary from the core of the species range to the periphery. For example, Green *et al*. [2] found allozyme diversity of *Rana pretiosa* was greatest at the center of the species range, corresponding to the peak model. In the gradient model, intraspecific variation changes from one end of a species range to the other, often corresponding to environmental variation. The gradient model has been demonstrated to apply to variation of both molecular markers and heat and cold tolerance phenotypes in *Drosophila melanogaster* [3], [4]. Few studies have addressed whether the gradient or peak model applies to plant intraspecific diversity. Richards *et al.* [5] hypothesized that intraspecific diversity would decrease in progressively more stressful environments for plant species able to reproduce clonally. However, they did not find any trend between diversity and environment. Genetic isolation by distance [6] may influence spatial genetic structure due to clonal growth, limited dispersal, genetic drift, selection, and density.

*Andropogon gerardii* (Vitman) is the dominant plant species in the North American tallgrass prairie ecoregion. Because of its importance in conservation and restoration ecology, and more recently in potential biofuel systems, the spatial genetic structure of *A. gerardii* has been an ongoing subject of interest [7], [8], [9]. Gustafson *et al.* [7] did not find a statistically significant relationship between random amplified polymorphic DNA (RAPD) similarity and geographic distance overall, though some prairies exhibited this trend. Most of the genetic variation (89%) was found within populations and 11% among populations. Gustafson *et al.* [8] also found that local prairie remnants and restored populations were genetically different than non-local remnants and cultivars. This suggests that the geographic origin of seed selected for restoration purposes may be important if the restoration goal is to produce a prairie plant community similar to the historical prairie at a particular site. Other restoration and conservation goals may include development of plant communities resilient to climate and land use change [10], which will require a much more complete understanding of the ecological genomics of species such as *A. gerardii* [11], [12],[13], including the role of disease resistance genes.

Plant disease resistance may vary across environmental gradients [14], [15], [16], contributing to the environmental gradient experienced by pathogens, but the population genetic structure of disease resistance genes across these gradients has rarely been addressed in natural plant populations [17], [18]. To our knowledge, no reports have been made of resistance genes or homologs varying in diversity across an environmental gradient distinct from overall genomic diversity across that gradient [17]. Roughly one percent of the protein-coding genes in plant genomes are disease resistance genes, with 207 predicted resistance genes in *Arabidopsis*, approximately 400 in *Populus*, and more than 500 in *Oryza* [19]. The importance of this class of genes in plant genomes is also demonstrated by the unusually high level of selection found at disease resistance loci [20], [21]. Burdon and Thrall [16] have made impressive progress in understanding the spatial dynamics of phenotypic resistance and pathogenicity in the natural flax-flax rust pathosystem. Within-population dynamics for disease resistance have also been addressed through modeling [22], [23], [24], [25], [26], [27]. However, typical coevolution model assumptions about costs and benefits of resistance for plants and virulence for pathogens, are often not appropriate for actual populations [26], [27]. Field studies of resistance gene diversity in natural populations have been helpful in identifying the various types of selection occurring at these loci [28] and indicating that different regions of resistance gene sequences are under different types of selection [29].

This study examines the diversity of a resistance gene homolog in *A. gerardii*. This species presents a unique opportunity to address questions about the distribution of resistance gene variation. It has a relatively continuous spatial distribution across a wide range of environments in the Great Plains and has common pathogens with its more-studied relative *Zea mays*. Understanding the ecology and evolution of disease resistance genes in such widespread and abundant native plant species could have direct applications to agriculture, where many crop species are grown in monocultures over large areas. The spatial structure of resistance genes influences epidemic intensity in many systems [30], and the effect of this spatial structure, itself, has the potential to vary across gradients [31]. Higher interspecific plant diversity has been observed to decrease disease in tallgrass prairie plants [32] and intraspecific resistance gene diversity has the potential to provide similar ecosystem services for disease regulation in *A. gerardii* populations [33]. Patterns of disease resistance variability in *A. gerardii* may also be important in the development of biofuel systems based on prairie plant species, including ‘low-input high-diversity’ systems [34].

No disease resistance genes have been cloned from *A. gerardii*, however we report the presence of a homolog of the maize gene *Rxo1* in *A. gerardii*. Zhao *et al.* [35] demonstrated that *Rxo1* confers resistance to *Burkholderia andropogonis* and a resistance reaction to the rice bacterial streak pathogen *Xanthomonas oryzae* pv. *oryzicola* after transfer to rice, suggesting conserved functionality across different genera of grasses. It is known that *Rxo1* recognizes avirulent *B. andropogonis* in maize, and that the same maize isolates of *B. andropogonis* infect *A. gerardii* [S. H. Hulbert, unpublished]. In addition, *Rxo1* recognizes not only avirulent *B. andropogonis*, but also *avrRxo1* in *Xanthomonas oryzae* pv. *oryzicola* [35], suggesting that *Rxo1* recognizes conserved components of a bacterial effector or group of effectors [36] or recognizes modifications to a cellular component that is a common effector target [37]. These results suggest that the *A. gerardii Rxo1* homolog may recognize avirulent *B. andropogonis* strains.

*Burkholderia andropogonis* is pathogenic to at least 51 plant species in 15 families of monocots and dicots [38]. It has been observed to cause low levels of bacterial stripe disease on *A. gerardii* at Konza Prairie Biological Station in Kansas under wet conditions in spring [39]. *B. andropogonis* infects sorghum and maize in warm and humid areas and is disseminated by splashing water [40]. Leaf surface moisture typically supports higher infection rates for bacterial foliar pathogens [38], [41], and bacterial stripe and leaf spot of maize caused by *B. andropogonis* tends to be more severe during periods of wet and warm weather [42]. Likewise, severity of bacterial brown leaf spot of citrus caused by *B. andropogonis* is associated with rainstorms in Florida [43].

The widespread distribution of *A. gerardii* across the North American Great Plains allows for the examination of resistance gene homolog diversity across an east to west gradient in precipitation. Net primary productivity increases by a factor of 5.5 from the Shortgrass Steppe Long Term Ecological Research Site in northeastern Colorado to the Konza Prairie Biological Station in Kansas [44]. Alexander *et al.* [45] found decreased disease (smut and rust) on *Carex blanda* field and herbarium specimens from western Kansas compared to eastern Kansas. This trend was attributed to the drier conditions in western Kansas providing an environment less suitable for the disease, the fact that western Kansas was at the edge of the range for *C. blanda*, and that western populations were smaller and more isolated, decreasing the potential for successful dispersal. Disease in *A. gerardii* caused by *B. andropogonis* likely also changes across this environmental gradient. The scale of selection for resistance can be relatively small. For example, differences in selection for herbivore resistance were found within a 4 ha stand of oak seedlings [46]. Variation in selection at the *Rxo1* homolog may result in different levels of diversity among populations of *A. gerardii*.

In this study, our first objective was to determine genome-wide diversity of *A. gerardii* across the U.S. Great Plains precipitation gradient using AFLP markers. We found novel evidence for spatial genetic structuring in *A. gerardii*, but not evidence for a change in genomic diversity across the gradient. Second, we assessed diversity in *Rxo1* homologs in the same natural populations of *A. gerardii*. We found that diversity in the resistance gene homologs decreased with decreasing precipitation across the gradient, in a pattern distinct from that of genomic diversity. The patterns of AFLP and *Rxo1* homolog diversity within and between prairies also provide perspectives on the spatial scale at which intraspecific variation occurs for both the entire genome and for a presumably adaptive gene family, as context for planning tallgrass prairie conservation and restoration.

## Materials and Methods

### Tissue Collection

**Figure 1:**
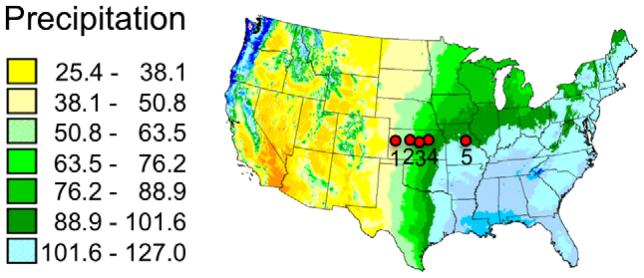
Location of prairies sampled across a significant precipitation gradient in the Great Plains. Prairie locations are (1) Smoky Valley Ranch, (2) Wilson Lake, (3) The Land Institute, (4) Konza Prairie Biological Station, and (5) Tucker Prairie. The background shading indicates an interpolation of annual average precipitation (cm) from 1961–1990 (where the precipitation map is copyright PRISM Climate Group, Oregon State University, http://prism.oregonstate.edu).

**Figure 2:**
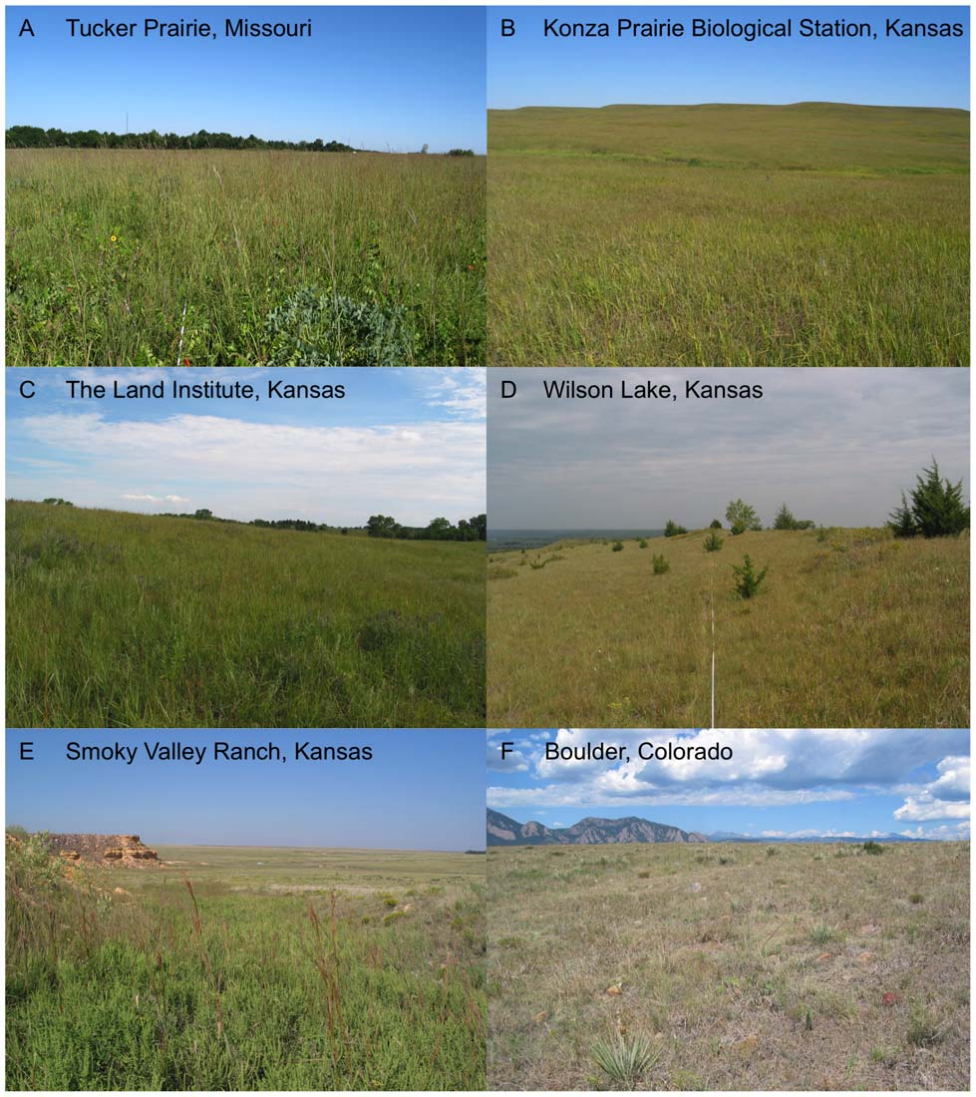
Collection sites for *Andropogon gerardii*.

**Figure 3:**
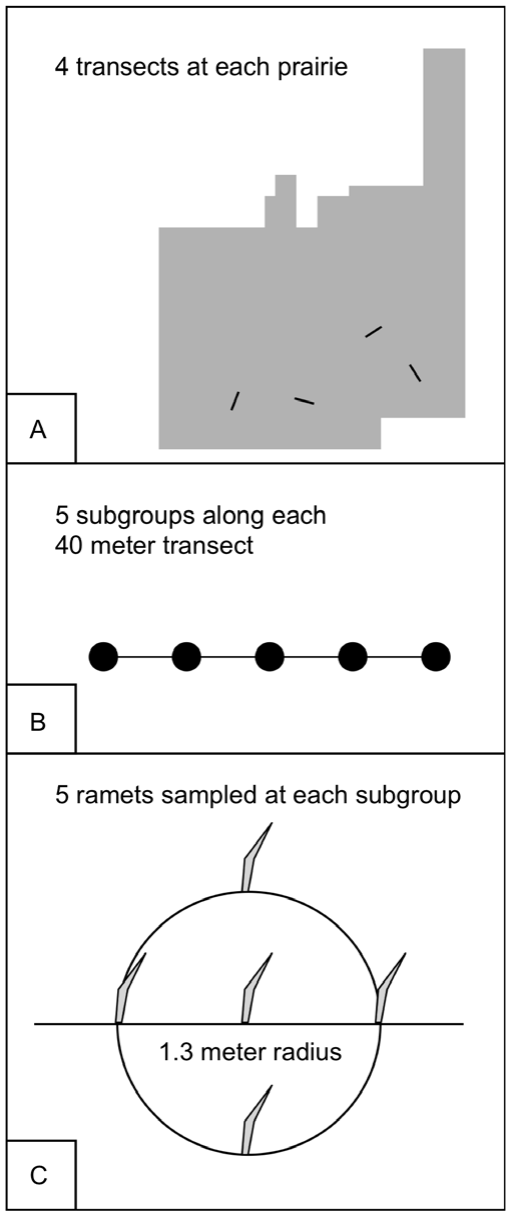
Sampling methodology at different scales. Each prairie (A) contained four transects. Each 40 m transect (B) contained a series of five subgroups. Five ramets were sampled at each subgroup (C).

Tallgrass prairie remnants can be found in much of the eastern US, but are most common in the Great Plains where a sharp east-west precipitation gradient occurs (Table 1; Fig. 1). We selected native tallgrass prairie locations at approximately the same latitude across this gradient (Table 1; Fig. 2). Within each prairie, four 40 m transects were established with five sampling points at 10 m intervals, for a total of 5 samples per point and 25 samples per transect (Fig. 3). Each ramet sample consisted of a 15 cm basal cutting made from two leaves placed in a sealed plastic bag with 15 ml of silica gel (Demis Products, Lithonia, GA and Miracle Coatings, Anaheim, CA) for storage up to one year at room temperature. One hundred tissue samples were collected from each prairie. Tissues were also collected from 65 clones in Boulder, Colorado, that had been mapped and characterized to ploidy level by Keeler [9] in plots 28, 52, 61, and 102 established by Jane and Carl Bock in the City of Boulder Open Space and Mountain Parks. DNA was extracted according to Doyle & Doyle [47] with several modifications (Appendix S1).

**Table 1:** Tallgrass prairies sampled, with state, UTM coordinates, AFLP sample size, *Rxo1*-RFLP sample size and average annual precipitation from the nearest weather station (1971–2000).

Prairie	State	UTM coordinates	AFLP sample size	*Rxo1*-RFLP sample size	Average annual precip. (cm/year)
Tucker Prairie	Missouri	15 N 587416 E, 4311575 N	71	100	104.7
KPBS^1^	Kansas	14 N 709536 E, 4327791 N	84	100	88.39
TLI^2^	Kansas	14 N 624616 E, 4292829 N	85	100	81.76
Wilson Lake	Kansas	14 N 527770 E, 4310686 N	70	100	65.18
Smoky Valley Ranch	Kansas	14 N 328103 E, 4306146 N	97	100	47.63
Boulder^3^	Colorado	13 N 475769 E, 4427696 N	63	63	50.62

1 Konza Prairie Biological Station.

2 The Land Institute.

3 Tissues from Boulder were used only in ploidy analyses.

### AFLP Genotyping

AFLP fingerprinting was performed according to Vos *et al.* [48] with many modifications (Appendix S1). Sixty-three repeatable AFLP polymorphic fragments were scored for the 6FAM-labeled *Eco*RI-AAA/*Mse*I-CTG primer-pair and 32 polymorphic fragments were scored for the HEX-labeled *Eco*RI-ACC/*Mse*I-CTG primer-pair. Capillary electrophoresis was used to identify fragments and GeneMarker version 1.6 software (SoftGenetics, State College, PA) was used to score AFLP markers. Statistical analyses described below were conducted using R software [49].

### *Rxo1* Homolog Identification

The sequences of the maize *Rxo1* gene and its four other tightly linked family members from the maize inbred B73 (GenBank accessions AY935244-AY935248) were compared to identify conserved sequences near the predicted start codon and nucleotide binding site (NBS) regions. Two conserved primers were designed from these sequences: ATG forward (5′-GAAGGGATGGCAGAGATTGCTGT-3′) and MHD reverse (5′-CAAGTTCACGCACAAGATCATGCAT-3′). These primers amplified fragments of approximately 1500 nt from genomic DNA of a single *A. gerardii* plant (clone B10, ploidy unknown) obtained from Konza Prairie Biological Station. Cloning and partial sequence analysis of these fragments identified three different sequences highly similar to the maize *Rxo1* gene which were then used to design RACE 5′ and 3′ PCR primers for 5′-TCTTTTTCAGGACAAGAACA-3′ and 5′-CGACGGAGCATGCTTCATGTTGCAGAGAG-3′ for 3′, respectively. Transcripts corresponding to the full coding region were then amplified with primers that were conserved among the RACE PCR amplicons; the primers 5′BRxo forward (5′-GGTATTGGGGCTAAAGAGTGAGCTTTGAC-3′) corresponded to sequences in the 5′UTR and 3′BRxo including the predicted stop codon (5′-GAGTTCTGCTATTTCATCTTAACCGGGCC-3′). RACE PCRs and cDNA amplifications were performed using the BD smart RACE cDNA amplification Clontech kit (Mountain View, CA). One cDNA corresponding to the full coding region had a 2793 nt open reading frame that was approximately 91% identical to the maize *Rxo1* coding region (Genbank sequence # pending).

### *Rxo1* Homolog Amplification and Restriction Enzyme Digestion

We amplified sequences from the leucine-rich repeat (LRR) encoding region from *A. gerardii* clones originating from diverse locations (Appendix S1). A primer pair was designed to amplify an 810 bp fragment (5′-AGATTCTCGACGAGTTGCTGTGCT-3′ and 5′-AGCCTAAGAAGCCCATTTCCGTGA-3′). The primers did not require pre-screening for polymorphism, and amplified fragments from plant samples collected throughout the gradient and beyond in Colorado, Illinois, and Indiana. Four restriction enzymes were used to perform RFLP assays: *BsaM*I, *Rsa*I, *Taq*I (Promega), and *Mse*I (New England Biolabs). The PCR amplicons were digested individually. Digestion products were electrophoresed on 2% agarose gels and scored for the presence or absence of fragments. Five fragments were scored for *BsaM*I, nine for *Mse*I, nine for *Rsa*I, and 14 for *Taq*I. We found the RFLP markers to be greater than 99% repeatable.

### Relatedness of Individuals of Different Ploidy

Like many perennial plant species, *A. gerardii* exists in populations with multiple ploidy levels [50]. Populations of mixed ploidy may complicate estimates of genetic diversity if the ploidy levels of individuals are unknown and genetic diversity varies with ploidy. *A. gerardii* populations consist predominantly of hexaploids and enneaploids with some individuals of intermediate ploidy levels [50], [51]. In general, hexaploids are more common in eastern prairies whereas western prairies (west of the Missouri River) have populations with mixed ploidy [50]. The origin and mechanism for maintenance of enneaploids is not known since they have not been found to have a reproductive fitness advantage in the field [52], at least in the short term, and crosses within or among any ploidy level very rarely yield enneaploid individuals [52], [39], [53]. In order to examine the distribution of resistance gene homologs in *A. gerardii*, it is necessary to account for the potential effects of spatial trends in ploidy. Population analyses of *A. gerardii* may be influenced by two factors. First, enneaploid and hexaploid individuals may be acting as separate populations. This could influence our results if enneaploids and hexaploids do not contribute equally to our diversity measures and if the proportion of enneaploids varies among prairies. Second, enneaploids may exhibit more AFLP amplicons compared to hexaploids because enneaploids contain 50% more chromosomes. In order to determine if enneaploids and hexaploids were acting as distinct populations, we measured AFLP dissimilarity among and within individuals of the two ploidy groups from the Boulder population. In order to test if enneaploid individuals were more diverse than hexaploid individuals we calculated the average number of AFLP fragments amplified in enneaploids and compared this to the hexaploid average. Assuming the polymorphic fragments analyzed in this study are distributed equally across chromosomes, we expected to find more fragments in enneaploids than in hexaploids. These ploidy analyses were conducted with data from the mapped Boulder population to determine if there is an inherent relationship between ploidy and diversity in *A. gerardii*.

### Dissimilarity and Geographic Distance

Scale of sampling is important in determining spatial autocorrelation in clonal species [54]. To test for spatial structure at different scales using the AFLP and *Rxo1*-RFLP datasets, a similarity matrix with an entry for each pairwise comparison of ramets from all prairies outside Colorado was calculated for each of the two datasets. To estimate pairwise genetic similarity for this study, we used the simple match coefficient (*s*), which Kosman & Leonard [55] argue is the most appropriate index for polyploid species, to calculate genetic similarity (the number of matches, in terms of shared absence or presence of a DNA fragment, divided by the total number of possible matches). Dissimilarity was calculated as one minus similarity (1-*s*). The mean dissimilarities of all ramet pairs at a particular distance from each other were used to calculate the overall mean dissimilarity for that distance. For example, since there were five plants in the first transect subgroup of a transect and five in the second transect subgroup, the mean of all of the comparisons among these two transect subgroups was used as one datum in calculating the overall 10 meter mean dissimilarity. Mean dissimilarity was calculated for approximately 1.8 m (average within-subgroup distance), 10 m, 20 m, 30 m, 40 m, 2139 m (average distance between transects), and 473,025 m (average distance between prairies). In order to test for spatial genetic structure, a regression was performed for mean dissimilarity versus distance between sampled locations. This test is similar to a Mantel test [56]. The test was performed with the AFLP marker data. We evaluated whether the slope parameter estimate was significantly greater than would be expected by chance by comparing it to parameter estimates from 1000 random permutations of the data (details in Appendix S1).

Shannon's information measure was used to determine the proportion of diversity among populations: *H′* = −Σ *p_i_* log *p_i_*, where *p_i_* is the frequency of the amplified fragment. *H′* was calculated for each population and used to determine the average population diversity: *H_a_*. The total diversity for all individuals used in this study was calculated and denoted as *H_s_*.

### Diversity and Precipitation

To determine the proportion of diversity among populations, the average population diversity was subtracted from the species diversity and this value was divided by the species diversity. This analysis was done with both AFLP and RFLP data. We chose two statistics to measure within-prairie diversity. First we calculated the average *Rxo1* and AFLP dissimilarity for each prairie. This measure of diversity includes haplotype diversity in addition to diversity of individual alleles since individual haplotypes were used in calculating dissimilarity. Shannon's information measure was also calculated for each population for both AFLP and *Rxo1* data. This measure of diversity considers allelic diversity alone. We used R to perform linear regression analyses of both *Rxo1* and AFLP diversity measures, with annual average precipitation (Table 1) as the predictor in separate analyses. Precipitation data for each prairie were estimated as the average annual precipitation from the nearest weather station between 1971 and 2000 [57].

## Results

### AFLP Dissimilarity and Ploidy

Samples collected in Boulder, Colorado, were from mapped clones of known ploidy. AFLP dissimilarity was calculated among hexaploid clones (n = 38), among eaneaploid clones (n = 21), and between hexaploid and enneaploid clones. The mean dissimilarity for each of these comparisons was found to be 0.10 indicating equal dissimilarity both within and among ploidy groups. There was no difference in average fragment number among ploidy levels. Average number of fragments for hexaploids was 59.37 (n = 38, 58.76–59.97 95% confidence interval) and 59.33 (n = 21, 58.25–60.42 95% confidence interval) for enneaploids. Similarly, the average number of *Rxo1*-RFLP fragments for hexaploids was 10.71 (n = 38, 10.59–10.83 95% confidence interval and 10.62 (n = 21, 10.41–10.83 95% confidence interval) for enneaploids.

### Dissimilarity and Geographic Distance

**Figure 4:**
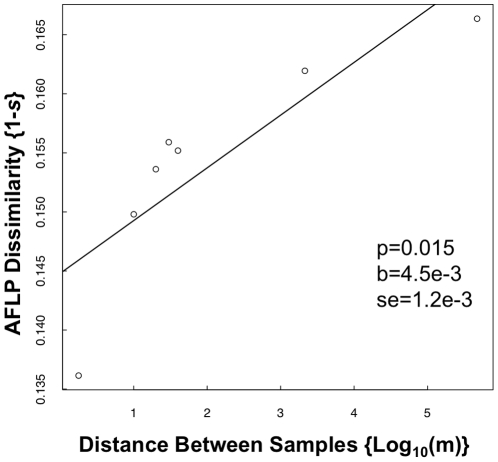
AFLP dissimilarity by geographic distance. Points from left to right are plotted at 1.8 m (within subgroup), 10 m (between adjacent subgroups), 20 m, 30 m, 40 m, 2139 m (between transects), and 473,025 m (between prairies). The slope parameter (‘b’), and 95% confidence interval from a randomization test are indicated.

Mean pairwise dissimilarity based on AFLP markers increased with increasing distance between individuals (Fig. 4). These data indicate the presence of spatial genetic structure in the *A. gerardii* populations (which may ultimately be described best by an asymptotic function). The trend of increasing dissimilarity with increasing distance was also significant using the *Rxo1-*RFLP data (data not shown). We found 6.35% of the AFLP variation to be present among populations based on Shannon's information measure. For *Rxo1*-RFLP data, 13.00% of the variation was distributed among populations.

### *Rxo1* Dissimilarity Relative to AFLP Dissimilarity

**Figure 5:**
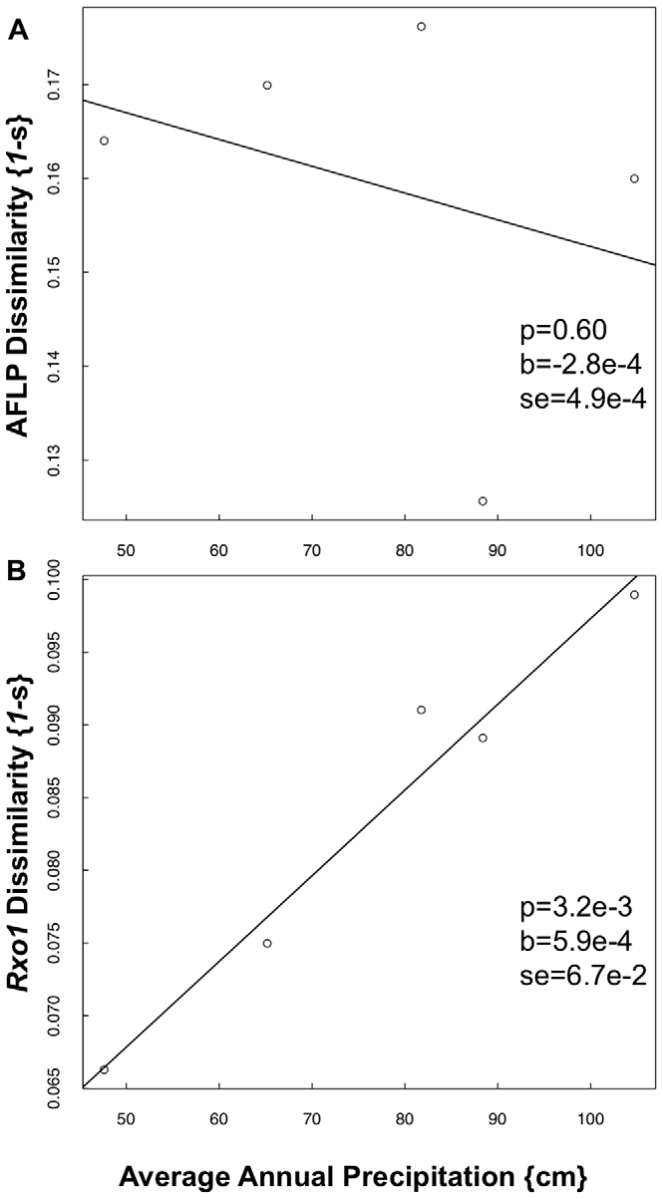
AFLP (a) and *Rxo1* (b) dissimilarity across precipitation. The mean dissimilarity for each prairie was calculated and plotted according to average annual precipitation. The p-value (‘p’), slope parameter (‘b’) and standard error (‘se’) from a linear regression model are indicated.

**Figure 6:**
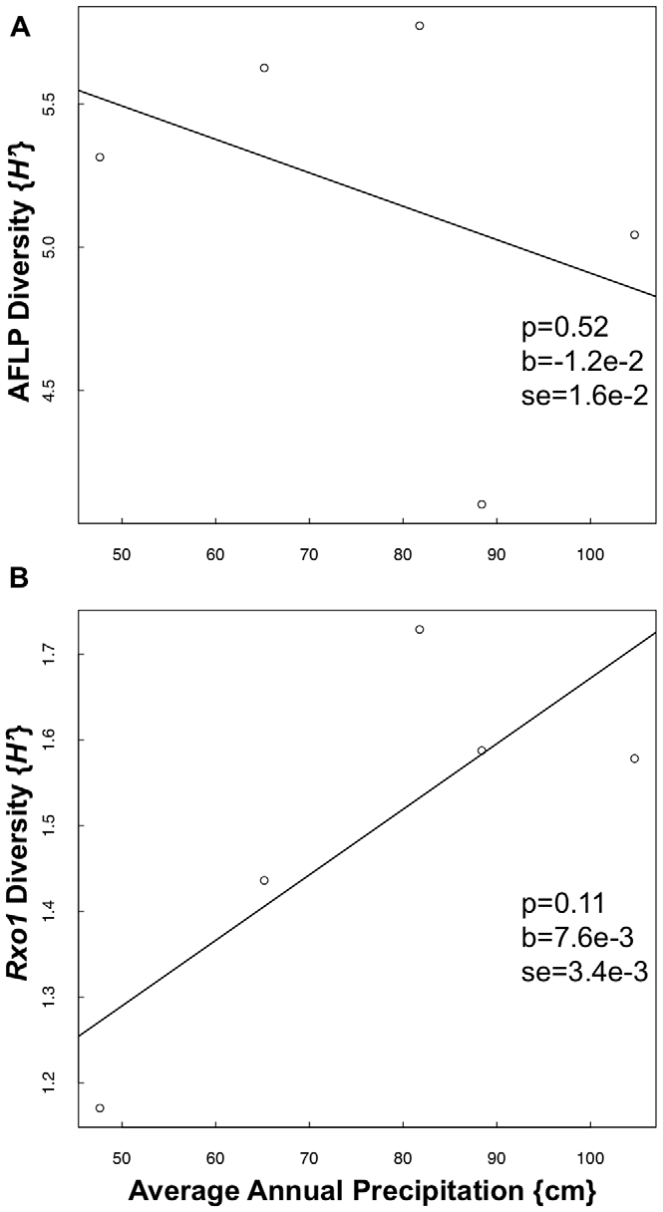
Shannon's information measure (*H′*) calculated for AFLP (a) and *Rxo1* (b) results for each prairie as a function of average annual precipitation. The p-value (‘p’), slope parameter (‘b’) and standard error (‘se’) from a linear regression model are indicated.

Mean *Rxo1* homolog dissimilarity must be examined in the context of mean AFLP dissimilarity to determine whether the pattern in dissimilarity across longitude for *Rxo1* is different from the pattern for the genome as a whole. We found a positive relationship between average annual precipitation and *Rxo1* homolog dissimilarity (Fig. 5). We did not find a significant relationship between AFLP dissimilarity and precipitation (Fig. 5). For Shannon's information measure, we found a similar trend with marginal significance between *Rxo1* diversity and precipitation, and no relationship between AFLP diversity and precipitation (Fig. 6). These data indicate that there is a positive relationship between *Rxo1* diversity and precipitation, and that this relationship is not due to a positive relationship between precipitation and genome-wide diversity, as measured by AFLP diversity.

## Discussion

A previous study of *A. gerardii* identified decreasing RAPD similarity with increasing distance within a subset of populations, though this trend was not consistent among prairies [7]. Our results demonstrate spatial genetic structuring among prairies. In our study, 6.3% and 13% of the AFLP and *Rxo1-*RFLP variation, respectively, were found among populations. Gustafson *et al.* [7] reported 11% of the variation was among prairies in Arkansas populations. The observed spatial genetic structure and estimated levels of population differentiation are significant for (1) validating the quality of the AFLP data and (2) providing preliminary data for decisions about minimum tallgrass prairie reserve acreage and strategies for selection of *A. gerardii* seed sources for prairie restoration. For the latter purpose, it will also be important to consider variation for genes under a range of types of relevant selection [11], [12].

There was a positive correlation between *Rxo1* homolog diversity and precipitation. This correlation was not simply the result of genome-wide influences on *A. gerardii*, because there was no trend in AFLP dissimilarity across the same gradient. In contrast, a geographic cline in *Cf-2* homolog diversity was consistent with a cline in diversity of neutral variation in natural populations of *Solanum pimpinellifolium* [17]. Information about which pathogen species and strains interact with this resistance gene locus in *A. gerardii* will be needed to fully assess the potential role of disease pressure in producing the gradient of *Rxo1* homolog diversity.

A compelling explanation for the *Rxo1* diversity cline is the presence of a gradient of disease pressure associated with the precipitation gradient. If a pathogen is found to interact with the *Rxo1* homologous locus (loci) in a coevolutionary manner, measurements of disease pressure in a common garden experiment of clones with known resistance levels across the precipitation gradient could be used to test the hypothesis that the diversity cline is associated with a gradient of disease pressure.

*Rxo1* homolog diversity was significantly higher in the eastern prairies. This may be due to selection for multiple alleles or gene family members in the disease-conducive environments, which would be consistent with the Red Queen hypothesis [58] applied to a plant-pathogen coevolutionary arms race [59], [60]. Diversifying selection characteristic of such an arms race has been observed in disease resistance loci that interact directly with pathogen effector proteins [61]. Alternatively, selection at loci with only single functional alleles can also promote stable polymorphisms if the functional alleles cause fitness disadvantages in environments with little disease [26]. Such fitness costs have been demonstrated experimentally for the *Rpm1* locus in *Arabidopsis* [62] and both functional and nonfunctional alleles are maintained across broad geographic regions [63], [64]. In summary, increased disease pressure in the east with associated diversifying or balancing selection is one explanation for the trend in *Rxo1* homolog diversity.

In the western prairies (Smoky Valley Ranch and Wilson Lake), drier conditions and more isolated plant populations would tend to result in lower disease pressure [41], [45] and dissimilarity in the *Rxo1* homologs was disproportionately low relative to the neutral markers. Pathogens may be shared between tallgrass prairie and agricultural systems (e.g., [65]), increasing the connectivity of host systems for pathogens [66], but the extent to which this is an important factor for *B. andropogonis* is not known. Though the higher degree of isolation experienced by *A. gerardii* populations in the west could contribute to a decrease in overall diversity, the AFLP markers did not indicate any significant east-west trend. The increased similarity in *Rxo1* homologs in the west could be due to selection for a single valuable allele or there could possibly be selection for a gene linked to *Rxo1* that is important under western environmental conditions. Selection has been found at low-variability quantitative trait loci caused by selective sweeps in drought and saline adapted *Helianthus annuus* populations in Utah [67]. However, many recombination events are likely to have occurred in such a natural population over long periods of time, buffering any such linkage disequilibrium between *Rxo1* and potential linked loci under selection. In summary, selection pressures in the western prairies including purifying selection is a second possible explanation for the observed trend of *Rxo1* homolog diversity across environments.

Comparative sequence analysis of the *Rxo1* homologous genes within and among natural populations of *A. gerardii* will allow assessment of what types of selection are controlling diversity at the *Rxo1* homolog locus. It has been demonstrated in models that coevolutionary clines may result from multiple effects [68]. And the question remains whether the cline in *Rxo1* diversity is adaptive. Sequence data for *Rxo1* homologs across the different populations could help to elucidate whether or not the diversity cline is conferred by adaptive evolution. However, a substantial effort would be required to sequence the potentially multiple *Rxo1* homologs in each individual. Functional analysis of the genes would also be valuable to more fully understand the molecular evolution of the *Rxo1* homologs.

*Andropogon gerardii* is often found in populations of mixed ploidy [50], and our results indicate that, based on AFLP and *Rxo1*-RFLP dissimilarities, there was no differentiation between individuals of different ploidy. This is consistent with allozyme data suggesting that individuals of dissimilar ploidy level from the same plot were more similar than individuals of the same ploidy level from different plots [69]. We anticipated that the average number of AFLP fragments in enneaploids would be greater than the average number of fragments in hexaploids, since enneaploids have 50% more genomic DNA and AFLPs are dominant markers. However, we found no difference. *Rxo1*-RFLP band number was also the same across ploidy levels. While it is possible that the relationship between ploidy and band number could differ among populations of *A. gerardii*, this finding in the Boulder population suggests that relative proportions of hexaploid and enneaploid ramets in the other populations was not important for determining dissimilarity and diversity in our analyses.

Our finding of no difference in band number between the two ploidy levels has implications for the origin and establishment of enneaploids in mixed ploidy populations. Since enneaploids were not found to have an increase in heterozygosity, our data suggest that enneaploids may be produced through second division restitution (SDR) of hexaploid gametes. This process may be mediated by temperature, as chromosome doubling has been observed at high temperatures for maize and other species of the tribe Triticeae [70], [71]. Such temperature-dependent polyploidy may explain the origin and maintenance of enneaploids and explain the adaptive role of populations in the west with mixed ploidy levels, since enneaploids seem to be more vegetatively fit in marginal environments [9] where heat stress is more common, providing the opportunity for increased chromosome doubling. Experiments testing the hypothesis of temperature-dependent ploidy levels during meiosis could identify a unique mechanism for population-level genomic diversity.

We have demonstrated that resistance gene diversity in *A. gerardii* varies with precipitation, one of the most important environmental drivers of plant disease. Diversity of resistance phenotypes has been shown to vary with ecoregion previously in the *Linum marginale* – *Melampsora lini* pathosystem, where higher host phenotypic diversity (significantly associated with population mean resistance) was present in hill populations compared to bog populations [72]. However, hill populations of *Linum marginale* also showed higher diversity at neutral allozyme loci and were significantly distinct from bog populations as measured by these neutral markers. Also, the proportion of *Aegilops tauschii* resistance to stem and leaf rust was found to vary greatly by region, with the highest resistance exhibited by populations adjacent to the Caspian Sea [73]. Our study is unique in demonstrating a cline in resistance gene homolog diversity in natural plant communities that is independent of genomic diversity.

Diversity of important genes in native populations may prove to be particularly valuable if selection pressures shift rapidly due to changing climate [74]. Unfortunately, the diversity within native populations may not be adequate to allow adaptive change to keep up with the rate of climate change in the twenty-first century, as is apparently the case for *Chamaecrista fasciculata* populations in the Great Plains [75]. Though we did not find that genomic diversity changes across a precipitation gradient (and thus the genomic results provide no support for a gradient model of diversity), we found that the gradient model applies to resistance gene homolog diversity, indicating the importance of individual loci for adaptation to an environmental gradient. Our novel method for quantifying disease resistance gene diversity for an organism with a complex genome could enable studies of genes known or hypothesized to be important in other natural systems for which few genetic tools are available. Further studies integrating population-level dynamics with evolutionary history for disease resistance genes in dominant natural plant species will also help to inform more effective deployment of resistance genes in biofuel and other agricultural systems.

## Supporting Information

Appendix S1 This appendix provides additional details about the methods.
(DOCX)
