# Supplementary material for: Genomic and Resistance Gene Homolog Diversity of the Dominant Tallgrass Prairie Species across the U.S. Great Plains Precipitation Gradient
Source: PLoS One. 2011 Apr 12;6(4):e17641. doi: 10.1371/journal.pone.0017641 (PMC3075248; doi:10.1371/journal.pone.0017641)
Supplement: Appendix S1 — This appendix provides additional details about the methods. (DOCX) [file pone.0017641.s001.docx]

**Appendix S1**

# DNA Extraction Extended Protocol

For each plant tissue sample, several 2-cm cuttings were placed in a 1.5 ml microcentrifuge tube and ground to a fine powder with a plastic peg under liquid nitrogen. Immediately after grinding, samples were placed in –20°C for up to 3 hours. Eight-hundred µl of 65°C 2× CTAB:β-mercaptoethanol buffer (99:1 v/v) were added to each tube and mixed with a pipette tip. The tubes were then inverted 10 times, placed in a 65°C water bath for 5-10 min., inverted 10 times again, and placed again in the water bath for 20-25 min. (30 min. total). Four-hundred µl of chloroform:isoamyl alcohol (24:1 v/v) were added to each tube and tubes were gently inverted for 2-3 min. Tubes were then placed in a microcentrifuge and spun at 13,400 ×g for 5 min. Five-hundred µl of the aqueous phase were then transferred to a clean microcentrifuge tube. DNA was precipitated by adding 500 µl of isopropanol to each tube. Each tube was then inverted 10 times and left at room temperature for 5-10 min. DNA was then pelleted by centrifugation at room temperature for 5 min at 9,300 ×g. The aqueous phase was decanted and the DNA pellets were dried for 5 min at room temperature. The DNA pellets were then dissolved in 600 µl of TE buffer (100mM Tris HCl pH 8.0 and 1mM EDTA pH 8.0) and were incubated at 4°C overnight. Tubes were shaken and briefly centrifuged to resuspend the DNA. Three-hundred µl of phenol:choloroform:isoamyl alcohol (25:24:1) were added to each tube and the tubes were shaken up and down by hand for few seconds. Tubes were then centrifuged at 13,400 ×g for 5 min at room temperature. Four-hundred-fifty µl of the aqueous phase were then transferred to a clean microcentrifuge tube. Four-hundred-fifty µl of chloroform:isoamyl alcohol (24:1) were added to each tube and the tubes were shaken up and down by hand for a few seconds. The tubes were then centrifuged at 13,400 ×g for 5 min at room temperature. Two-hundred-fifty µl of the aqueous phase were then transferred to a clean microcentrifuge tube and one µl of RNAse A (Sigma-Aldrich, 2639 kunits/ml) was added to each tube. Tubes were shaken and briefly centrifuged before being incubated at 37°C for 30 min. DNA was precipitated by adding 250 µl of isopropanol to each tube. Tubes were then inverted 10 times and left for 5-10 min at room temperature. Tubes were then centrifuged at 9,300 ×g for 10 min at room temperature. The aqueous/alcohol mixture was decanted and DNA pellets were washed with 1 ml of 70% ethanol. Tubes were then centrifuged at 9,300 ×g for 5 min. The ethanol was decanted and DNA pellets were left to dry at room temperature for 10 minutes. The DNA pellet was dissolved in 50 µl of TE buffer and tubes were placed in 4°C overnight. Tubes were then gently flicked and briefly centrifuged to resuspend the DNA. DNA was assayed in 1% agarose gel submerged in 0.5× TBE buffer using known DNA concentrations of λ-DNA digested with *Hind*III (New England Biolabs). DNA concentrations were determined using a Gel-Doc EQ gel-reading system (Bio-Rad Laboratories, PA, USA).

# AFLP Extended Protocol

All primers were ordered from Integrated DNA Technologies (Coralville, IA, USA). The digestion and ligation reactions were conducted in a 21 µl reaction volume and had the following components: 2 µl genomic DNA, 0.25 µl 12 u/µl *Eco*R1 (Promega, Wisconsin, USA), 0.16 µl 10 u/µl *Mse*1 (New England Biolabs), 0.27 µl 3 u/µl T4 DNA ligase (Promega), 2 µl 10× H buffer (Promega), 2 µl ligase buffer (Promega), 0.4 µl *Eco*R I adapter mix (5 ρm/µl 5'-CTCGTAGACTGCGTACC-3' and 5 ρm/µl 5'-AATTGGTACGCAGTCTAC-3'), 0.4 µl *Mse*1 adapter mix (50 ρm/µl 5'-GACGATGAGTCCTGAG-3' and 50 ρm/µl 5'-TACTCAGGACTCAT-3'), and 13.52 µl sterilized distilled water (sdH_2_O). The digestion-ligation reactions were left overnight at 25°C. The preamplification reaction had the following components in a 40 µl reaction volume: 10 µl diluted (1:10) digestion-ligation mixture, 8 µl 5× PCR buffer (Promega), 4 µl 25mM MgCl_2_, 1.6 µl 2mM dNTPs, 0.76 µl 100ng/µl *Eco*R1-A primer (5'-AGACTGCGTACCAATTCA-3'), 0.76 µl 100ng/µl *Mse*1-C primer (5'-GATGAGTCCTGAGTAAC-3'), 0.15 µl 5 u/µl Gotaq Flexi DNA Polymerase (Promega,), and 14.73 µl sdH_2_O. The preamplification reactions were performed in a MJ Research PTC-200 thermocycler (MJ Research Inc., Watertown, MA, USA) with 1 min. at 94°C followed by 30 cycles of 30 seconds at 94°C, 1 min. at 56°C, and 1 min. at 72°C. Sixteen primer pairs were tested for the selective amplification step. Out of the 16 primer pairs, two (*Eco*R1-AAA/*Mse*1-CTG and *Eco*R1-ACC/*Mse*1-CTG) were selected because they demonstrated the most polymorphism for plant samples collected from across our entire study gradient and into Colorado, Illinois, and Indiana. *Eco*R1-AAA was labeled with the fluorescent dye 6FAM and *Eco*R1-ACC was labeled with the fluorescent dye HEX (Integrated DNA Technologies, Inc.). The selective amplification reactions were performed with the following components in a 20.5 µl reaction volume: 1.5 µl diluted (1:20) preamplification reaction, 4 µl 5× PCR buffer (Promega), 2 µl 25mM MgCl_2_, 2 µl 2mM dNTPs, 2 µl 50 ng/µl *Eco*R1-selective primer, 3 µl 50 ng/µl *Mse*1-selective primer, 0.2 µl 5 u/µl Gotaq Flexi DNA Polymerase (Promega), and 5.3 µl sdH_2_O. The PCR cycling conditions of selective amplification reactions were 2 min. at 95°C followed by 13 cycles of 30 seconds at 65°C (-0.7°C per cycle), 90 seconds at 72°C, and 30 seconds at 94°C, followed by 23 cycles of 30 seconds at 94°C, 30 seconds at 56°C, and 90 seconds at 72°C, and, followed by 5 min. at 72°C. The two selective amplification products labeled with 6FAM and HEX were diluted and combined in 96-well plates as follows: 1 µl *Eco*R1-AAA/*Mse*1-CTG selective amplification,1 µl *Eco*R1-ACC/*Mse*1-CTG selective amplification, 4 µl sdH_2_O, 8.8 µl formamide, 0.2 µl GeneScan 500 Liz size standard (Applied Biosystems, Foster City, CA, USA). The mixture was then incubated in a thermocycler for 5 min. at 95°C. The 96-well plates were then submitted to the USDA Small Grain Genotyping Laboratory (Manhattan, Kansas) for AFLP analyses using ABI 3100 DNA Analyzer (Applied Biosystems). AFLP data were analyzed using GeneMarker version 1.6 with the manufacturer’s suggested settings except that the “smooth” option was selected and “reject” and “check” options for peak evaluation were set to zero.

# AFLP Repeatability

Seven replicates of 15 DNA extracts were run separately through the AFLP protocol in order to estimate repeatability of the AFLP profiling. AFLP peaks with low repeatability were removed from the final analyses. We found AFLP peak repeatability to be 90.6%. Much of the variation in repeatability of peaks was due to one or two replicates of each DNA extract being disproportionately different compared to the rest of the replicates (data not shown). Overall, replicates of the repeatability test, run on the same plate as random arrangements of other samples, resulted in poorer quality peaks than other samples (data not shown). This may be due to more frequent handling and thawing for the samples that were repeatedly analyzed. Therefore, the 90.6% band repeatability average is likely an underestimate of the band repeatability for most samples.

Additional Sampling of *Rxo1* Sequence Polymorphism

Forward (F) and reverse (R) primer pairs were designed from conserved regions of the *A. gerardii* or maize *Rxo1* sequence as follows: 1F= 5'-CTCCTGAGTTACGTCAGTGTG-3'; 1R = 5'-CAGTGTCTTCAAAGCTGCACGC-3'; 2F = 5'-GGCCATGCAGCTTAGAAGAC-3'; 2R = 5'-ATCGAGGCACAAAAGCCTAA-3'; 3F = 5'-GCAGAGAGGAACAGCTTTGG-3'; 3R = 5'-CCCCTGTGGGAACTTCACTA-3'; 4F = 5'-GGAAACAATGAGGCAATGCT-3'; 4R = 5'-AGGAACCAGTCTGCTTGGAA-3'; 5F = 5'-TTCCTGCAAACCGAAGTACC-3'; 5R = 5'-TTCCCTTTTGAATGCTGCTT-3'. PCR amplifications were purified using the QIAquick PCR purification kit (Qiagen, MD, USA); cloned with the Qiagen PCR CloningPlus kit. Five to ten clones selected from each cloned PCR amplicon were sequenced. DNA sequencing was performed by the USDA Small Grain Genotyping Laboratory (Manhattan, Kansas). DNA sequence alignments were performed using BioEdit software (http://www.mbio.ncsu.edu/BioEdit/BioEdit.html) and polymorphisms in restriction sites were examined to identify the restriction enzymes for the PCR-RFLP step. *A. gerardii* plant tissues were sequenced from across the precipitation gradient and also to the east and west of the gradient (Illinois, Indiana, and Colorado). Selection of restriction enzymes from sequences derived from this geographic expanse avoids ascertainment bias.

## PCR-RFLP

The PCR amplifications were performed in 25 µl reaction volume as follows: 1 µl genomic DNA, 4 µl 5× PCR buffer (Promega), 2 µl 25mM MgCl_2_, 2.5 µl 2mM dNTPs, 1 µl 25 µM forward primer, 1 µl 25 µM reverse primer, 0.25 µl 5 u/µl Gotaq Flexi DNA Polymerase (Promega), and 13.25 µl sdH_2_O. The PCR conditions were as follows: one cycle at 95°C for 5 min. followed by 30 cycles of 30 seconds at 95°C, 30 seconds at 60°C, and 90 seconds at 72°C, followed by 1 min. at 72 C.

# Dissimilarity and Geographic Distance

Following the null hypothesis, the plant identification numbers were randomized along the axis of the similarity matrix and means were calculated again for each distance for each randomization. The plant identification numbers were randomized instead of the similarity values in the matrix to preserve the relevant structure of the dataset. One thousand permutations yielded 1000 means under this null hypothesis for each distance. We used the 25th and 976^th^ ordered means as the lower and upper 95% confidence intervals for dissimilarity at each distance under this null hypothesis of no difference in dissimilarity at any distance. This method was used for separate analyses of both the RFLP and AFLP similarity matrices. If the observed mean for a particular distance was above or below the confidence interval, that mean was considered significantly greater or lesser than expected by chance under the null hypothesis, indicating nonrandom population structure.
